# Differential Effects of Social and Health Factors across the World: A Comparison between African American and Indigenous African Cohorts of the effects of Sociality, Clinical Health, and Sex factors on Cognition and Alzheimer's Disease

**DOI:** 10.1002/alz70860_103662

**Published:** 2025-12-23

**Authors:** Joseph M Curran, Jasmit Shah, Cynthia Isabel Smith, Kathleen A. Lane, Sujuan Gao, Michelle M Mielke, Adesola Ogunniyi, Hugh C Hendrie, Chinedu T Udeh‐Momoh

**Affiliations:** ^1^ Division of Public Health Sciences, Wake Forest University, School of Medicine, Winston‐Salem, NC, USA; ^2^ Aga Khan University, Nairobi, Kenya; ^3^ Brain and Mind Institute, Aga Khan University, Nairobi, Nairobi, Kenya; ^4^ Indiana University School of Medicine, Indianapolis, IN, USA; ^5^ Institute for Advanced Medical Research and Training, College of Medicine, University of Ibadan, Ibadan, Nigeria; ^6^ Brain and Mind Institute, Aga Khan University, Nairobi, Kenya

## Abstract

**Background:**

Given the prominence of WEIRD (Western, Educated, Industrialized, Rich, and Democratic) samples in the field public health sciences, there is limited research on risk factors of cognitive impairment, dementia, and Alzheimer's disease (AD) in underserved populations. This is especially present in Africa, where academic research has largely neglected the continent, leading to a lack of longitudinal studies seeking to understand dementia and Alzheimer's.

**Methods:**

Using data from the Indianapolis‐Ibadan Dementia Project, a cross‐national longitudinal study of cognition, clinical health, and dementia risk among African Americans in Indianapolis (*n* = 3,982) and Indigenous Africans in Ibadan, Nigeria (*n* = 4,353), we examined the effects of social factors, clinical health, and sex on AD development and on cognitive domains (specifically semantic memory, episodic memory, and executive functioning).

**Results:**

5.8% of participants developed Alzheimer's at follow‐up. Of the modifiable factors evaluated, we anticipate that individuals with higher social engagement will have lower odds of being diagnosed with AD at follow‐up, and higher cognitive scores across both the African American and Indigenous African cohorts. We will also evaluate the impact of comorbidities—defined as the presence of two or more co‐occurring medical conditions—for predicting higher AD risk and lower cognitive scores across both cohorts. Additionally, we propose that the above relationships will be modified by sex at birth, being stronger for females compared to males, in line with preliminary analysis showing higher risk of dementia and AD for African women.

**Conclusions:**

Our findings contribute to a cross‐cultural understanding of the cultural, health, and social factors that influence AD risk and cognitive domain scores across diverse populations of African ancestry, providing a framework for tailoring interventions and treatments for AD that are informed by cultural factors.

